# Aging atlas reveals cell-type-specific effects of pro-longevity strategies

**DOI:** 10.1038/s43587-024-00631-1

**Published:** 2024-05-30

**Authors:** Shihong Max Gao, Yanyan Qi, Qinghao Zhang, Youchen Guan, Yi-Tang Lee, Lang Ding, Lihua Wang, Aaron S. Mohammed, Hongjie Li, Yusi Fu, Meng C. Wang

**Affiliations:** 1 Janelia Research Campus, Howard Hughes Medical Institute, Ashburn, VA USA; 2 Program in Developmental Biology, Baylor College of Medicine, Houston, TX USA; 3 Huffington Center on Aging, Baylor College of Medicine, Houston, TX USA; 4 Molecular and Cellular Biology Graduate Program, Baylor College of Medicine, Houston, TX USA; 5 Integrative Program of Molecular and Biochemical Science, Baylor College of Medicine, Houston, TX USA; 6 Graduate Program in Chemical, Physical & Structural Biology, Graduate School of Biomedical Sciences, Baylor College of Medicine, Houston, TX USA; 7 Department of Biomedical Sciences, Creighton University School of Medicine, Omaha, NE USA; 8 Department of Molecular and Human Genetics, Baylor College of Medicine, Houston, TX USA

**Keywords:** Ageing, RNA sequencing

## Abstract

Organismal aging involves functional declines in both somatic and reproductive tissues. Multiple strategies have been discovered to extend lifespan across species. However, how age-related molecular changes differ among various tissues and how those lifespan-extending strategies slow tissue aging in distinct manners remain unclear. Here we generated the transcriptomic Cell Atlas of Worm Aging (CAWA, http://mengwanglab.org/atlas) of wild-type and long-lived strains. We discovered cell-specific, age-related molecular and functional signatures across all somatic and germ cell types. We developed transcriptomic aging clocks for different tissues and quantitatively determined how three different pro-longevity strategies slow tissue aging distinctively. Furthermore, through genome-wide profiling of alternative polyadenylation (APA) events in different tissues, we discovered cell-type-specific APA changes during aging and revealed how these changes are differentially affected by the pro-longevity strategies. Together, this study offers fundamental molecular insights into both somatic and reproductive aging and provides a valuable resource for in-depth understanding of the diversity of pro-longevity mechanisms.

## Abstract (web summary)

This comprehensive resource offers new insights into how different types of cell and tissue change with age in *C. elegans* and unveils the distinctive anti-aging effects of various pro-longevity strategies in a cell-type-specific manner.

## Main

For multicellular organisms, aging affects the functions of all somatic and reproductive tissues. How age-related molecular changes differ in various tissues at cellular resolution remains poorly understood. In addition, although multiple pro-longevity strategies have been discovered in multicellular organisms ranging from *Caenorhabditis elegans* to mice^1–12^, whether and how these strategies slow aging of different tissues in distinct manners are yet to be determined. In recent years, single-cell and single-nucleus RNA sequencing (scRNA-seq and snRNA-seq) have proven to be effective ways to systemically profile transcriptomes at single-cell resolution and have facilitated the discovery of cell-type-specific transcriptomic signatures in different tissues^13–22^. It was also shown that snRNA-seq is less biased for tissue sampling in atlas studies compared to scRNA-seq, because certain cell types (for example, muscle and epidermal cells) cannot be efficiently isolated using single-cell dissociation methods^23,24^. In this study, we used snRNA-seq transcriptomic profiling of different somatic and germ cell types to build an adult cell atlas. Using snRNA-seq data from wild-type (WT) adults at different ages, we generated tissue-specific transcriptomic aging clocks as well as germ cell differentiation trajectory maps to assess how aging affects the function of different cell types. We also revealed age-associated, tissue-specific transcriptomic changes associated with three different pro-longevity mechanisms. Furthermore, we profiled pre-mRNA alternative polyadenylation (APA) at the genome level in different cell types at different ages and systemically discovered APA events with tissue-specific patterns and how age-associated APA changes in different tissues are attenuated by those pro-longevity mechanisms. To openly share this resource, we developed a user-friendly data portal to visualize data.

## Results

### Developing snRNA-seq platform for adult worms

**Fig. 1:**
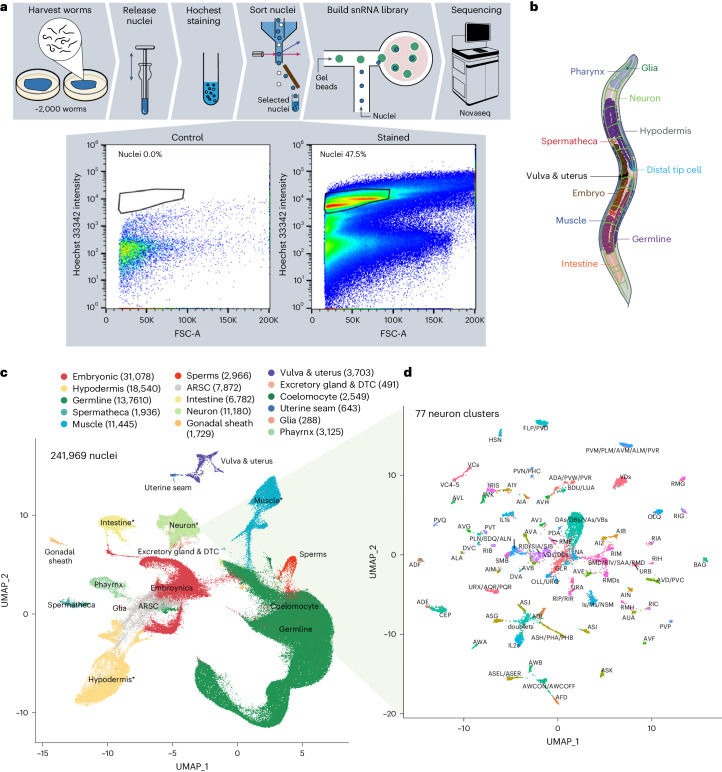
Adult *C. elegans* cell atlas at single-cell resolution. **a**, Schematics of single-cell transcriptome profiling pipeline in adult *C. elegans*. The process begins with harvesting and homogenizing approximately 2,000 worms to isolate nuclei. These nuclei are subsequently stained with Hoechst dye and sorted through FACS to select for positively stained nuclei. FACS gating graphs based on Hoechst staining intensity show a clear separation between intact nuclei and debris, ensuring the quality of subsequent snRNA-seq analyses. The selected nuclei are then used to build an snRNA library to conduct next-generation sequencing. **b**, Anatomical illustration of an adult *C. elegans*, detailing major tissues. **c**,**d**, UMAP plot visualization of 241,969 single nuclei from adult *C. elegans* cell atlas. Colored clusters correspond to 15 major tissues and ARSC. The numbers in parentheses are the numbers of nuclei in each tissue. DTC, distal tip cells. Tissues marked with * can be further subclustered. Seventy-seven subsets of neurons are shown in **d** and others in Extended Data Fig. 1.

**Extended Data Fig. 1:**
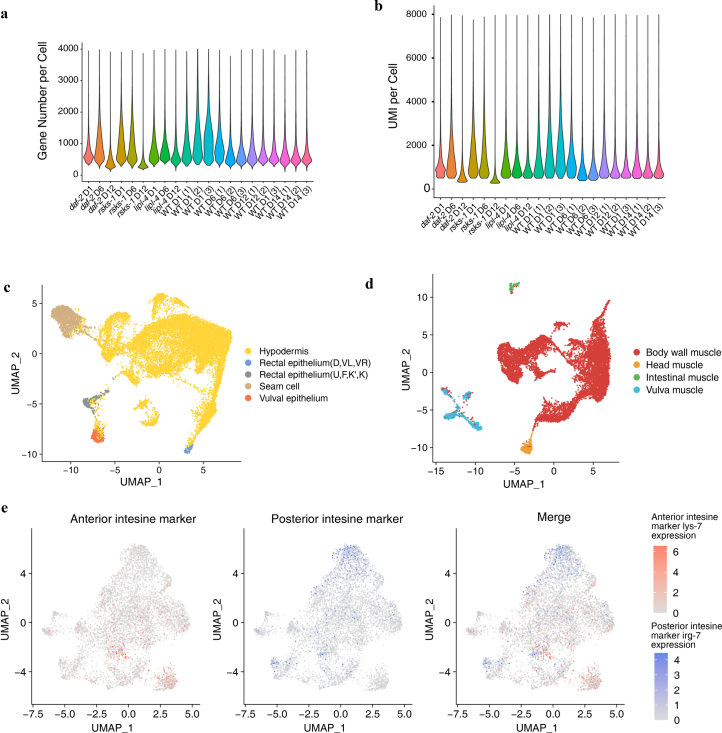
Details of snRNA-seq workflow and tissue sub-clustering. **a** and **b**, Gene numbers and unique molecular identifiers (UMI) per cell are consistent between experiments. **c**, UMAP visualization of hypodermis sub-clustering showing seam cell, rectal epithelium, and vulva epithelium. **d**, UMAP visualization of muscle sub-clustering into body wall muscle, intestinal muscle, head muscle, and vulva muscle. **e**, UMAP visualization of intestine sub-clustering showing marker gene expression separation of the anterior (*lys-7*) and posterior (*irg-7*) region.

We developed an snRNA-seq pipeline for systemically profiling transcriptomic changes in adult *C. elegans* at single-cell resolution (Fig. 1a). For each experiment, we harvested and homogenized approximately 2,000 worms. Nuclei were isolated using fluorescence-activated cell sorting (FACS) based on the DNA content signal (Fig. 1a), and snRNA-seq was performed using the 10x Genomics platform. For each experiment, 10,000 nuclei were sequenced to capture the transcriptome of 959 somatic cells and approximately 2,000 germ cells in adult *C. elegans*. The mean gene count and unique molecular identifiers per cell were consistent among all experiments, which are 906 and 1,490, respectively (Extended Data Fig. 1a,b). After pre-processing and cell filtering, we generated 241,969 single-nuclei gene expression profiles. We first combined all samples from different genotypes and ages to achieve a more reliable cell type annotation, like in other atlas studies^21,25^. From this dataset, we built an adult cell atlas that covers 15 major cell classes, including neurons, glia, hypodermis, intestine, muscle, pharynx, coelomocyte, gonadal sheath cells, vulva and uterus, uterine seam cells, distal tip cells and excretory gland cells, germline, sperms, spermatheca and embryonic cells (Fig. 1b,c and Supplementary Table 1). The subclustering of these major cell classes further revealed more different cell types within each class. For neurons, 77 subclusters were identified, which account for 104 of all 118 adult neuron classes (Fig. 1d)^26^. For the hypodermis, we distinguished seam cells and rectal and vulval epithelium from other hypodermal cells (Extended Data Fig. 1c). For the muscle, we discovered four subclusters, including body wall muscle, head muscle and vulva muscle (Extended Data Fig. 1d). For the intestine, we identified subclusters that likely correlate with the anterior and posterior regions based on known gene expression markers (Extended Data Fig. 1e).

### Revealing cell-type-specific molecular and functional signatures

**Fig. 2:**
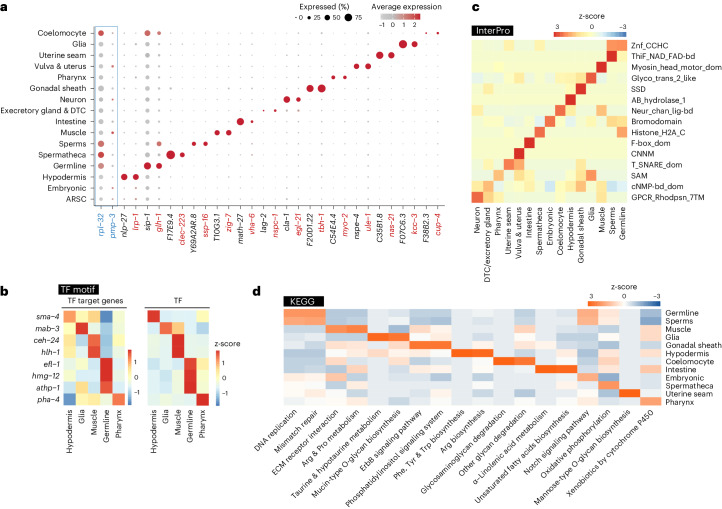
Systemic view of cell-type-specific transcriptional and functional landscape. **a**, Dot plot visualizing the cell-type-specific expression pattern of housekeeping genes (blue) and known (red) and newly identified (black) gene markers for each major tissue. Housekeeping genes *rpl-32* and *pmp-3* show pan-cell expression. Dot size corresponds to the percentage of cells within a specific tissue expressing the marker gene, and the color intensity indicates the average expression level of the gene across the tissue. **b**, Heatmaps showing the expression levels of the tissue-specific TFs (right) with the corresponding enrichment scores of their target genes (left) in each tissue. **c**,**d**, Heatmaps of tissue-specific functional modalities selected based on the enrichment scores using InterPro (**c**) and KEGG (**d**) pathway analyses. cNMP-bd_dom, cyclic nucleotide-binding domain; CNNM, cyclic nucleotide-binding domain; F-box_dom, F-box domain; Histone_H2A_C, histone H2A, C-terminal domain; Neur_chan_lig-bd, neurotransmitter-gated ion-channel ligand-binding domain; SAM, sterile alpha motif domain; SSD, sterol-sensing domain; T_SNARE_dom, target SNARE coiled-coil homology domain; ThiF_NAD_FAD-bd, THIF-type NAD/FAD binding fold; Znf_CCHC, zinc finger, CCHC type.

These large-scale profiling and clustering analyses revealed cell-type-specific transcriptional signatures that are supported by previously identified gene markers (Fig. 2a). We also identified a series of new cell-type-specific gene markers, which showed similar or better specificity than well-known markers (Fig. 2a and Supplementary Table 1). Through DNA binding site motif exploration for transcription factor (TF) targets (Methods), we identified TFs that could mediate cell-type-specific gene expression, including *sma-3* (hypodermis), *mab-3* (glia), *ceh-24* and *hlh-1* (muscle), *efl-1*, *hmg-12* and *atph-1* (germline) and *pha-4* (pharynx) (Fig. 2b). Accordingly, their expression exhibited the same tissue specificity (Fig. 2b).

Next, we used InterPro and KEGG classification to analyze these cell-type-specific transcriptome profiles and discovered distinct functional features for each cell type. Some of these functional features are highly expected. For example, InterPro uncovered the specific enrichment of the G protein-coupled receptor family (GPCR, rhodopsin-like, 7TM (GPCR_Rhodpsn_7TM), key molecular sensors) and the myosin head motor domain family (myosin head, motor domain (Myosin_head_motor_dom), required for muscle contraction) in neurons and the muscle, respectively (Fig. 2c). The KEGG analysis revealed that alpha-linolenic acid metabolism and unsaturated fatty acid biosynthesis categories are enriched in the intestine responsible for fat storage (Fig. 2d) and DNA replication and mismatch repair categories in the germline with active cell division (Fig. 2d). Previously unknown tissue-specific functional signatures were also uncovered. For example, in glia, we discovered the specific enrichment of glycosyltransferase 2-like family (glycosyltransferase 2-like (Glyco_trans_2_like)) and mucin-type O-glycan biosynthesis based on InterPro (Fig. 2c) and KEGG (Fig. 2d), respectively, which together suggest the importance of O-glycosylation in glial physiology. We also found that the α/β hydrolase superfamily (alpha/beta hydrolase fold-1 (AB_hydrolase_1); Fig. 2c) and Arginine, Phenylamine, Tyrosine and Tryptophan biosynthesis (Fig. 2d) are specifically enriched in the hypodermis, indicating the active involvement of this tissue in metabolic processes.

**Extended Data Fig. 2:**
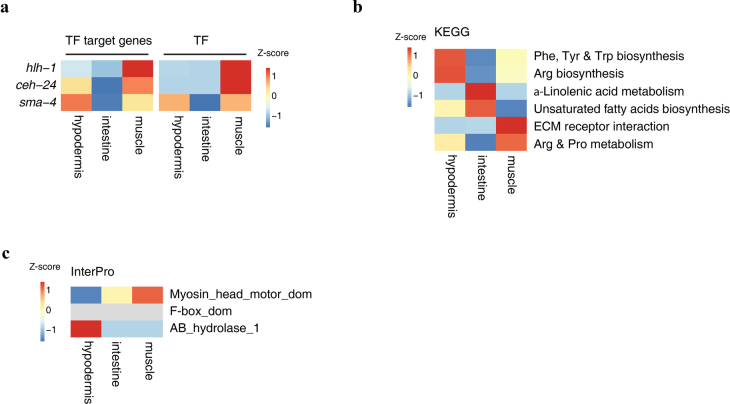
External validation of cell-type-specific molecular and functional features. **a**, Heatmaps showing the expression level of transcription factors (right) with the corresponding enrichment score of their target genes (left) enriched explicitly in tissues shown, based on the scRNA-seq dataset from the Roux et al study^21^. **b** and **c**, Heatmaps showing tissue-specific functional modalities based on Roux et al.’s scRNA-seq data^21^, represented by the enrichment score based on KEGG (**b**) and InterPro (**c**) pathway terms. The InterPro term ‘F-box_dom’ is represented in gray, indicating no expression for all seven genes in this term in the Roux et al. data.

We performed the same analyses using the recently published scRNA-seq datasets from *C. elegans* sterile mutants^21^. For the TF enrichment analysis, we observed consistent patterns in muscle, intestine and hypodermis (Extended Data Fig. 2a). We also identified the similar KEGG and InterPro enrichments (except for F-box domain containing seven genes, which were absent in the scRNA-seq dataset) for the tissues that were also annotated in the scRNA-seq dataset (Extended Data Fig. 2b,c).

### Building aging cell atlas under physiological conditions

**Fig. 3:**
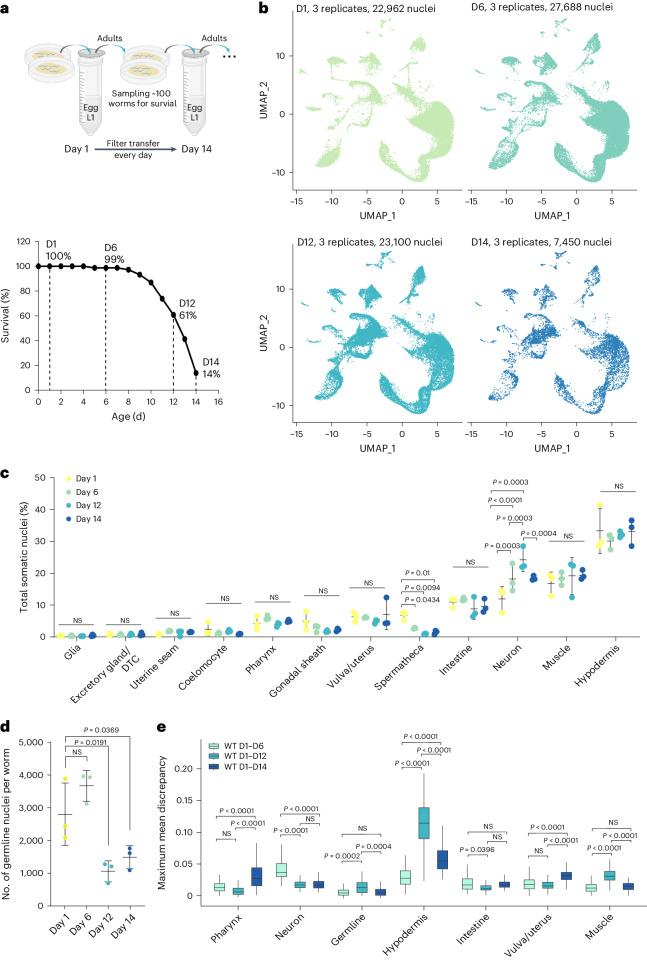
Aging cell atlas under physiological conditions. **a**, Schematic of aging sample preparation under physiological conditions without interrupting worm reproduction and the survival curve of worm samples used for nuclei collection at four timepoints to build the aging cell atlas. **b**, Cell atlases from four age groups shown by UMAP, three replicates per age group with total nuclei numbers. **c**, The percentage of various somatic cell types in total captured cells does not change much between four ages, except for spermatheca and neurons. Not significant (NS) *P* > 0.05. *P* values by one-way ANOVA test with Benjamini–Hochberg correction. *n* = 3 biologically independent samples for each timepoint. Data are presented as mean ± s.d. **d**, Numbers of germ nuclei decreased from day 1 and day 6 to day 12 and day 14. NS *P* > 0.05. *P* values by one-way ANOVA test with Benjamini–Hochberg correction. *n* = 3 biologically independent samples for each timepoint. Data are presented as mean ± s.d. **e**, Box plots displaying maximum mean discrepancy between age groups across different tissues in WT worms. NS *P* > 0.05. *P* values by one-way ANOVA test with Benjamini–Hochberg correction. *n* = 300 independent iterations. The box plot’s box spans the interquartile range (IQR), with the bottom and top representing the 25th and 75th percentiles, respectively, and the median value at the middle. Whiskers extend to the smallest and largest values within 1.5 times the IQR from the quartiles. D, day.

**Extended Data Fig. 3:**
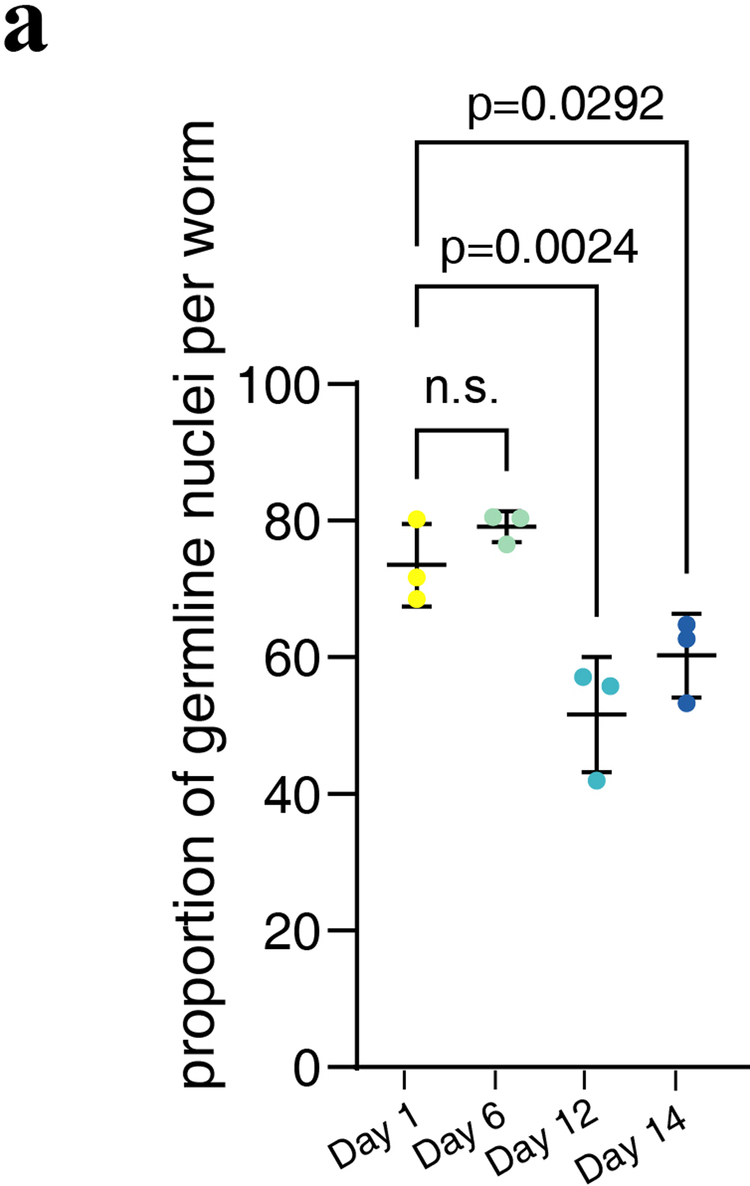
Age-related germ cell changes. **a**, Percentage of germ nuclei per worm decreases from day 1 and day 6 to day 12 and day 14. n.s. *P* > 0.05. *P* value by one-way ANOVA test with Benjamini–Hochberg correction. *n* = 3 biologically independent samples for each time point. Data are presented as mean values +/− SD.

Next, we focused on establishing the aging cell atlas to understand age-related transcriptomic changes in different cell types. We analyzed data from WT worms at four different adult ages, day 1, day 6, day 12 and day 14, when the survival rate was 100%, 99%, 61% and 14%, respectively (Fig. 3a), and built aging cell atlases (Fig. 3b). We found that the relative number of cells in different somatic tissue clusters remains similar during the aging process, except for spermatheca and neurons (Fig. 3c), which confirms that our snRNA-seq pipeline did not introduce sampling bias in most cell types. However, we observed that the number of cells in the germline cluster (Fig. 3d) and the percentage of germ cells among all cells (Extended Data Fig. 3a) decrease with aging.

Our aged samples were prepared without interfering with normal reproduction, which enabled us to investigate tissue-specific transcriptomic changes during both somatic and reproductive aging. Using the scMMD (maximum mean discrepancy) method, we first calculated the distance drift in the transcriptome of various tissues from day 1 to day 6, day 1 to day 12 and day 1 to day 14 in WT. It is worth noting that the analysis using day 6 samples captured almost the entire population, given the 99% survival rate at this age (Fig. 3a). However, by day 12, approximately 39% of the population had died (Fig. 3a), and, thus, the transcriptome analysis of the remaining survivors may introduce bias toward longer-lived individuals. This potential bias may become even more pronounced on day 14 when 89% of the population was dead (Fig. 3a).

Transcriptome drifts (>0.02) were observed in the hypodermis and neurons from day 1 to day 6, suggesting that these two tissues may be more sensitive to aging compared to others. Interestingly, the drift observed in neurons decreased on day 12 and day 14 (Fig. 3e), and a possible explanation is that individuals with more pronounced neuronal transcriptome changes might have died from the population at these later timepoints. The transcriptome drift in the hypodermis kept increasing until day 12 but did not further elevate on day 14 (Fig. 3e). The transcriptome drift in the muscle was increased on day 12, which was suppressed on day 14 (Fig. 3e). On the other hand, the pharynx and vulva/uterus exhibited a transcriptome drift only on day 14 (Fig. 3e). In contrast, the intestine did not show obvious drifts even on day 14 (Fig. 3e), indicating its transcriptome robustness during aging. These results suggest that neurons may be critical sites for delaying the onset of aging at an early stage, and hypodermis and muscle could be crucial for slowing down aging at later stages.

### Mapping germ cell fate trajectories during aging

We did not detect a transcriptome drift in the germline using scMMD analysis. Unlike post-mitotic cells in the soma, germ cells undergo proliferation and differentiation along the germline, which would result in aging-irrelevant transcriptome changes. Thus, developing distinct analysis platforms is needed for systemically analyzing transcriptome changes in the germline during aging. To this end, we chose to map germ cell trajectories first and then determine whether and how the trajectory pattern shifts during aging.

**Fig. 4:**
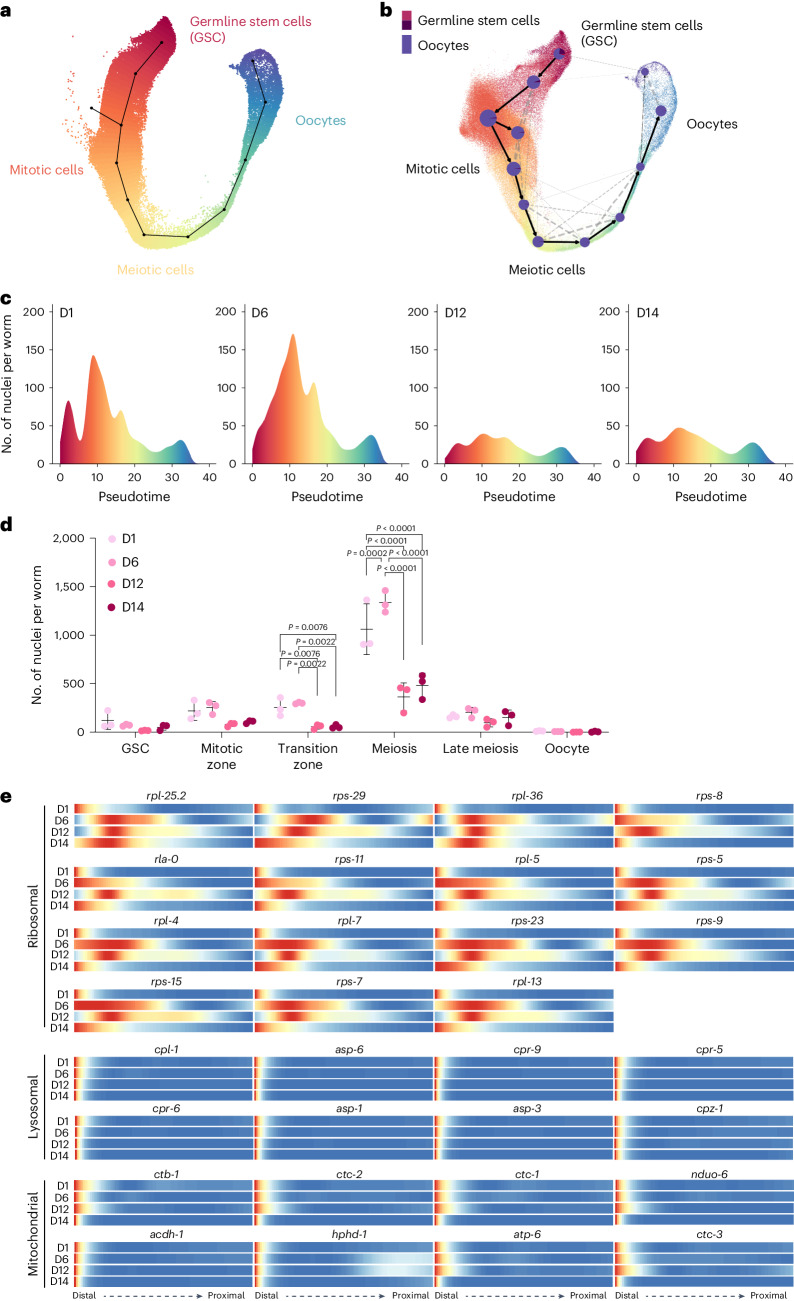
Germline trajectory mapping age-related changes. **a**, Trajectory pseudotime clusters germ nuclei into different cell identities. **b**, Germ cell trajectory PAGA map showing cell fate commitment from GSCs to mature oocytes. **c**, Density plots showing the numbers of germ nuclei distributed along the pseudotime at different ages. **d**, Strip chart showing the numbers of germ nuclei in different regions of the germline at different ages. *P* values by one-way ANOVA with Benjamini–Hochberg correction. *n* = 3 biologically independent samples for each timepoint. Data are presented as mean ± s.d. **e**, Heatmaps showing gene expression levels along the distal-proximal axis of the germline. Ribosomal, lysosomal and mitochondrial genes with distal-restricted expression on day 1 undergo age-related changes in different patterns.

**Extended Data Fig. 4:**
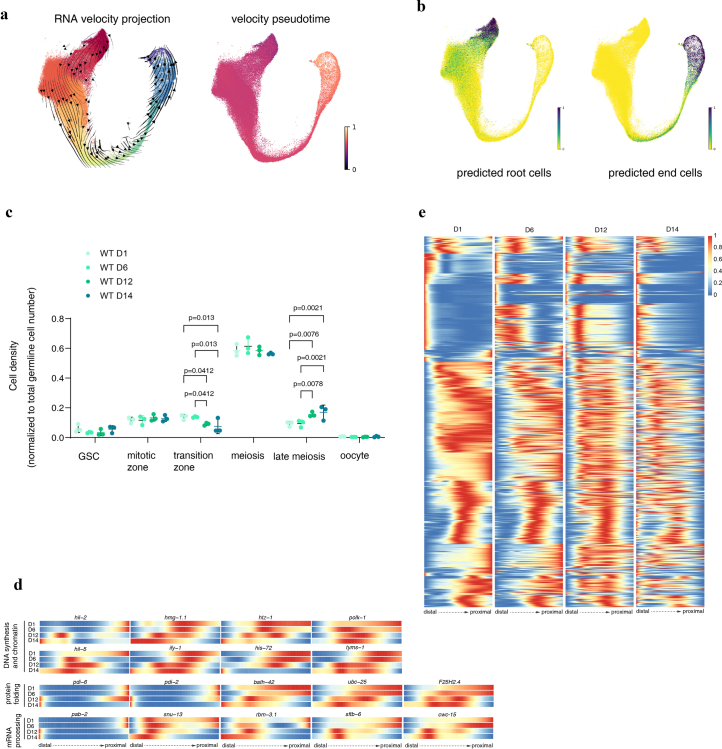
Germline trajectory mapping to identify age-related changes. **a**, Velocity projection graph in UMAP embedding and Velocity pseudotime. **b**, UMAP graph highlighting predicted root and endpoints that correlated with germline stem cells and oocytes, respectively. **c**, Strip chart showing the percentage of germ nuclei in different regions of the germline at different ages. * *P* <0.05, one-way ANOVA test with Benjamini–Hochberg correction. *n* = 3 biologically independent samples for each time point. Data are presented as mean values +/− SD. **d**, Heatmaps showing that gene expression temporal patterns along the developmental progression of germ cells are disrupted with aging. **e**, Heatmaps showing gene expression levels along the distal-proximal axis of the germline. Genes related to DNA synthesis, chromatin assembly, protein folding and degradation, and mRNA processing show age-related decrease in the proximal end of the germline.

We used two different computational methods, the Slingshot package^27^ and the RNA velocity-based scVelo algorithm^28^, to minimize analysis bias. The results obtained from two methods were consistent with each other. These trajectory maps depicted the progression of germ cells, as they undergo from germline stem cells (GSCs), through mitotic cells and meiotic cells, toward mature oocytes (Fig. 4a,b and Extended Data Fig. 4a). The unsupervised scVelo model computed the initial and terminal states within the trajectory and identified cell clusters at the root and at the endpoints, which correspond to GSCs and mature oocytes, respectively (Extended Data Fig. 4b). Furthermore, directed partition-based graph abstraction (PAGA) provided a quantitative assessment of cell fate probabilities for the initial, intermediate and terminal states (Fig. 4b), which is consistent with the developmental order from GSCs to fully differentiated oocytes.

The computational trajectory allowed us to construct a pseudotemporal order and visualize age-related changes in different regions of the germline (Fig. 4c). To quantitively analyze these changes, we assigned germ nuclei into separate regions, including GSC, mitotic zone, transition zone, meiosis, late meiosis/apoptosis and oocyte, based on their pseudotemporal orders and the previously annotated germline compartmentation^29^. We observed that the numbers of germ nuclei in the transition zone and meiosis region decrease from day 6 to day 12, with no further decrease at day 14 (Fig. 4d). We also calculated the percentage of germ nuclei in different regions and found decreases in the transition zone on day 12 and day 14 compared to day 1 and day 6 and increases in the late meiosis/apoptosis region (Extended Data Fig. 4c). These results suggest that the composition of the germline undergoes changes as organisms age.

To gain a molecular insight into these age-related changes, we identified hundreds of genes that display specific expression patterns within different germ cell groups and change their expression patterns during aging (Extended Data Fig. 4d; top ~600 genes listed in Supplementary Table 2 and Methods). A series of ribosomal protein-encoding genes exhibits restricted expression in the distal end of the germline on day 1 (Fig. 4e), consistent with the previous finding^29^. Interestingly, with increasing age, the expression of these ribosomal protein genes expands toward the proximal region starting on day 6 (Fig. 4e). It was reported that, in mutants lacking germ cell differentiation, ribosomal protein genes extend their expression from the distal end to the proximal end of the germline^29^. Thus, the age-associated proximal expansion of ribosomal protein gene expression may be associated with decreased germline differentiation during aging, underlying the decrease in the transition zone and the meiosis region (Fig. 4d).

Additionally, we found that genes encoding lysosomal proteinases exhibit restricted expression in the distal end of the germline, but their levels decrease with increasing age (Fig. 4e). The distal expression of genes encoding mitochondrial components was also decreased on day 14 (Fig. 4e). These results suggest declined organelle biogenesis in the distal germline during aging. At the proximal end of the germline, we observed an age-associated transcriptional decrease in genes related to DNA synthesis and chromosome assembly, protein folding and degradation and mRNA processing (Extended Data Fig. 4e), which may underlie the reduced quality of aged oocytes. Together, these analyses reveal the molecular signatures for germ cells at various stages and provide a comprehensive view of their alterations during aging.

### Developing tissue-specific aging clocks

**Fig. 5:**
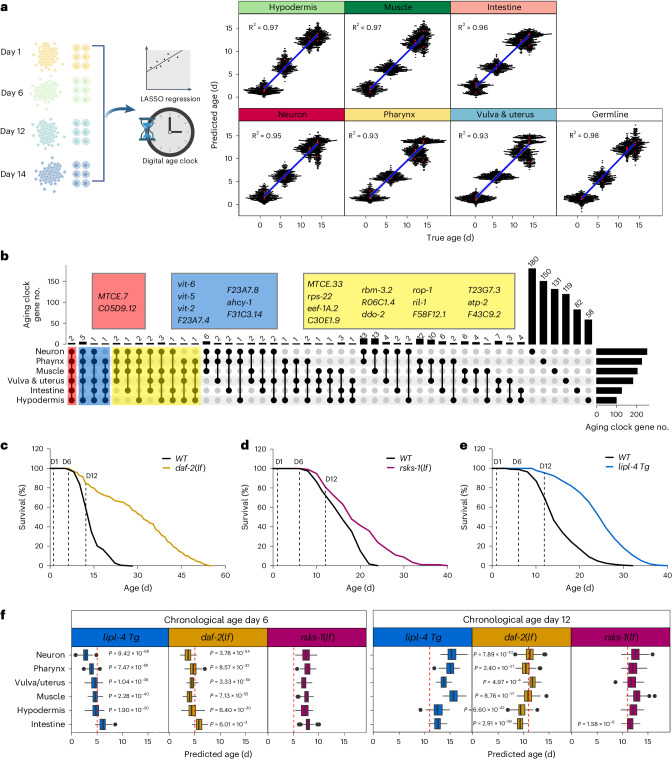
Aging clocks reveal tissue-specific anti-aging effects of different pro-longevity strategies. **a**, The schematic outlines the development of machine-learning-based tissue-specific transcriptomic aging clocks, which can predict the biological age of each tissue based on sn-RNAseq data from four timepoints (left). The performance of tissue-specific aging clocks validated with leave-one-batch-out cross-validation. Red dots represent median prediction for the test dataset, the blue line represents the fitted linear model through the prediction points, and the light gray area indicates the 95% confidence interval. The square of Pearson’s correlation coefficients is shown (right). **b**, UpSet plot showing the intersection sets of aging clock genes identified across tissues. Genes shared among six, five or four of the tissue-specific aging clocks are listed in red, blue or yellow boxes, respectively. **c**–**e**, Lifespans of *daf-2* loss-of-function mutant (*daf-2(lf)*) (**c**), *rsks-1* loss-of-function mutant (*rsks-1(lf)*) (**d**) and *lipl-4* transgenic strain (*lipl-4 Tg*) (**e**) compared to WT. **f**, Box plots showing the predicted biological ages of different tissues in 3 long-lived strains at the chronological ages of day 6 and day 12, as determined by tissue-specific aging clocks. Day 5 and day 11 indicated by red dashed lines present the cutoff for slowing down the clocks. One-sample *t*-test, one-sided, *n* = 100 BootstrapCells cells. *P* values are shown in the figure. For box plots, the center is the median; the lower and upper bounds correspond to the first and third quartiles, the whiskers extend up to 1.5 times the IQR and the minima and maxima are the observed minima and maxima. The box plot’s box spans the IQR, with the bottom and top representing the 25th and 75th percentiles, respectively, and the median value at the middle. Whiskers extend to the smallest and largest values within 1.5 times the IQR from the quartiles.

In parallel, we leveraged tissue-specific transcriptomic changes during aging to build age prediction models—aging clocks—for different tissues. Using machine learning, we constructed least absolute shrinkage and selection operator (LASSO) regression-based tissue-specific aging clocks, which is one of the top-performing chronological aging clocks^30^ and can accurately predict true chronological ages of tissues with more than 50 cells (Fig. 5a). To evaluate the performance of the aging clocks and avoid inflation, we applied the leave-on-batch-out cross-validation scheme. During the training, we dropped one set of biologically independent samples and used the remaining two sets of replicates with no shared worms for building the model. After training, we applied the aging clocks to predict the ages of the dropped samples. Our models predicted true chronological age with correlations (*R*^2^) greater than 0.90 for multiple tissues (Fig. 5a), which validated the prediction accuracy at all timepoints.

**Extended Data Fig. 5:**
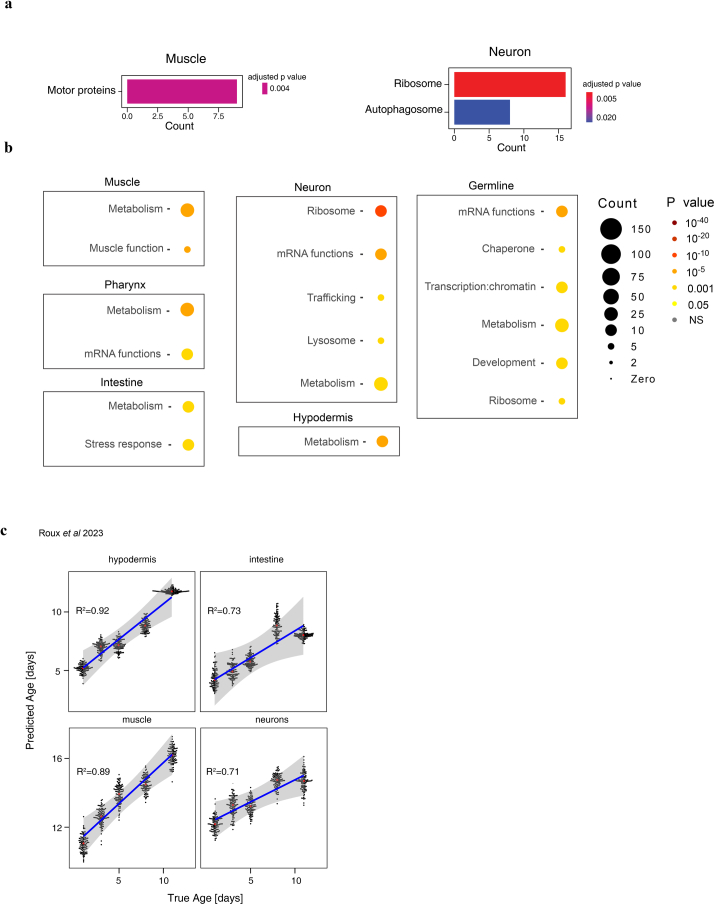
Functional enrichment and validation for tissue-specific age clocks. **a**, KEGG pathway enrichment analysis for aging-clock genes of muscle and neuron. **b**, WormCat gene ontology enrichment for ageing-clock genes of major tissues. **c**, Tissue-specific transcriptomic aging clocks validation with sc-RNAeq data from Roux et al. Jitter plots showing the correlation between the chronological age of the published scRNAseq data from germ free worms and predicted age with the corresponding tissue-specific aging clocks trained on WT worms. Red dots represent median prediction for the test dataset, blue line represents the fitted linear model through the prediction points, with light gray area indicating the 95% confidence interval. The square of Pearson’s correlations coefficients is shown.

We further identified genes that contribute to the prediction, terming them aging clock genes (Supplementary Table 2). These aging clock genes are mostly tissue specific and exhibit very little overlap (Fig. 5b). The only two shared genes among all tissues, *MTCE.7* and *C05D9.12*, are ribosomal RNA and non-coding RNA, respectively. When conducting KEGG analysis, neuron-specific aging clock genes displayed a significant enrichment in ribosome and autophagosome-related pathways, and muscle-specific aging clock genes were enriched in motor protein pathways (Extended Data Fig. 5a). With WormCat gene set enrichment analysis^31^, we found metabolism as an enriched term in all tissues except for vulva/uterus (Extended Data Fig. 5b). However, these metabolic genes show little overlap (Supplementary Table 2). These results suggest that different tissues age differently with their unique transcriptional signatures.

Additionally, we used the scRNA-seq dataset^21^ for external validation of tissue-specific aging clocks. The annotation of four somatic tissues, including neurons, hypodermis, intestine and muscle, was matched between the two studies. The predicted ages based on our tissue-specific aging clocks exhibited a good linear correlation with the actual ages (intestine (*R*^2^ = 0.73), hypodermis (*R*^2^ = 0.92), muscle (*R*^2^ = 0.89) and neurons (*R*^2^ = 0.71)) (Extended Data Fig. 5c) but with a drift. We noticed that the numbers of genes detected in the sc-RNAseq dataset were lower, and the absence of tissue-specific aging clock gene expression in many cells could contribute to the observed drift. Moreover, the scRNA-seq samples were collected from sterile mutants at a higher temperature (25 °C), which can accelerate the aging process. As a result, at the same chronological age, the biological age of worms at 25 °C will be older than that at 20 °C, leading to a drift in the age prediction.

### Determining tissue-specific anti-aging effects of pro-longevity strategies

For analyzing tissue-specific transcriptomic changes associated with different lifespan-extending mechanisms, we used the loss-of-function (lf) mutant of *daf-2* that reduces insulin/insulin-like growth factor signaling (IIS) and doubles the lifespan (Fig. 5c)^32^; the *lf* mutant of *rsks-1* that encodes S6 kinase downstream of TOR signaling and shows 20–30% lifespan extension (Fig. 5d)^8^; and the *lipl-4* transgenic strain (*lipl-4 Tg*) that induces lysosomal lipolysis and extends lifespan by 40–60% (Fig. 5e)^11^. snRNA-seq analysis was performed at three different ages (day 1, day 6 and day 12). We first used the tissue-specific aging clocks to predict the biological ages of six different tissues in these long-lived strains at the chronological age of day 6 and day 12. At day 6, the *daf-2(lf)* mutants and the *lipl-4 Tg* strains exhibited biological ages younger than 6 d (at least 1-d difference) for all tissues except for the intestine (Fig. 5f). The neuron is the youngest tissue with a biological age of 2.8 d in the *lipl-4 Tg* strain and 3.7 d in the *daf-2(lf)* mutant. However, in the *rsks-1(lf)* mutant, none of the tissues showed a predicted age younger than day 6 (Fig. 5f). At day 12, only the *daf-2(lf)* mutant still showed a biological age of 11 d or younger in all tissues except for vulva/uterus (Fig. 5f).

### Molecular regulation of tissue aging by different pro-longevity mechanisms

**Fig. 6:**
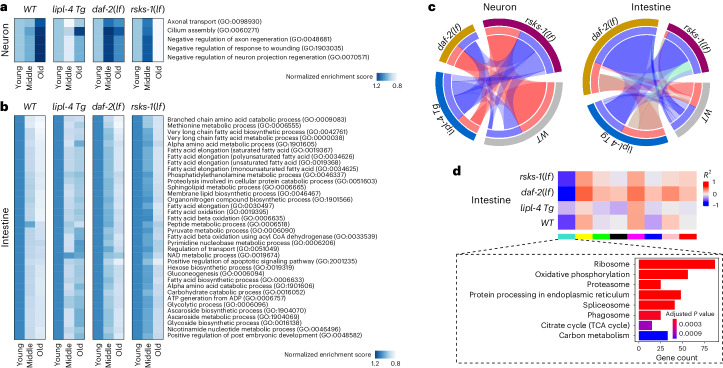
Effects of different pro-longevity strategies on tissue-specific age-related features. **a**,**b**, Tissue-specific age-related GO terms for neurons (**a**) and intestine (**b**) exhibit distinct patterns of changes in the three long-lived strains. The enrichment scores for each GO term were normalized to the young group. **c**, Circos plots showing conserved co-expression modules (Fisher’s exact test, *P* < 0.01) that were significantly correlated with aging (Pearson’s correlation, *P* < 0.001 and *R*^2^ > 0.2) in neurons (left) and the intestine (right) between different genotypes. Blue ribbons connect conserved models that were both negatively correlated with aging in different genotypes, red ribbons connect conserved models that were both positively correlated with aging and green ribbons connect conserved models that were oppositely correlated with aging in different genotypes. **d**, Correlations between consensus co-expression modules and aging in the intestine. Gene modules demonstrating significant correlation with aging (Pearson’s correlation, *P* < 0.001 and *R*^2^ > 0.2) in at least one genotype are shown. Bar graphs enclosed in dashed boxes represent KEGG pathways that were significantly enriched (Fisher’s exact test, Benjamini–Hochberg-adjusted *P* < 0.01) for the turquoise module in the intestine.

**Extended Data Fig. 6:**
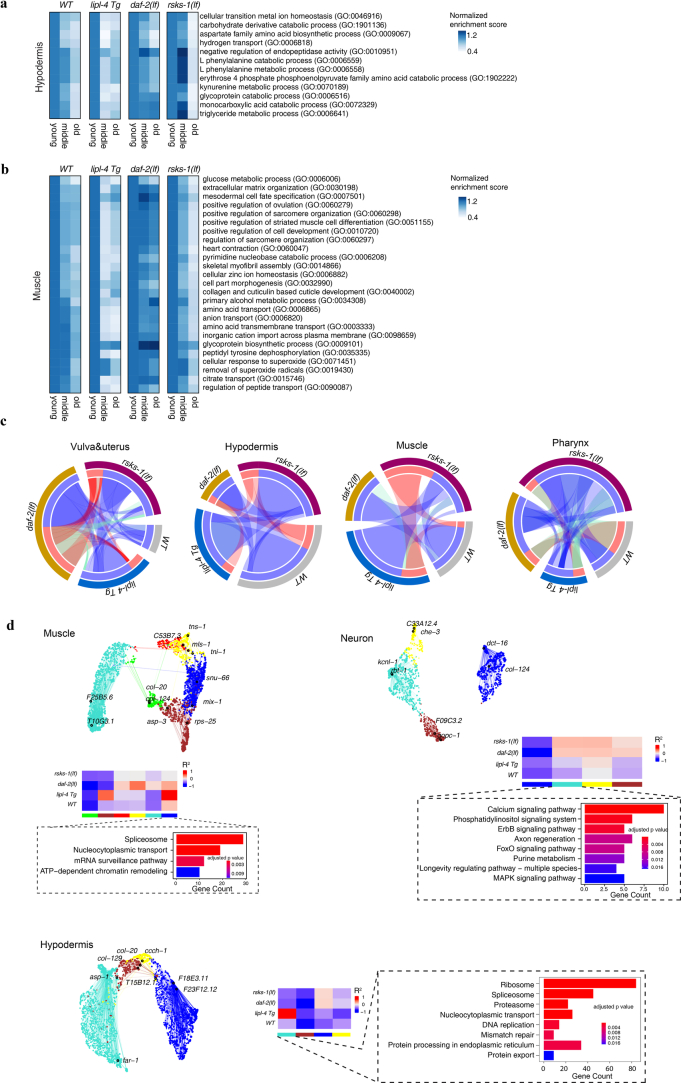
Age-related molecular regulations of tissue-specific aging by different pro-longevity mechanisms. **a** and **b**, Heatmaps show that tissue-specific significant age-related Gene Ontology (GO) terms for hypodermis (**a**) and muscle (**b**) exhibit distinct patterns of changes in the three long-lived strains. The enrichment scores for each term were normalized to the young group. **c**, Circos plots showing conserved co-expression modules (Fisher’s exact test, *P* < 0.01) that are significantly correlated with aging (Pearson’s correlation, *P* < 0.001 and *R*^2^ > 0.2) in vulva and uterus, hypodermis, muscle, and pharynx between different genotypes. Blue ribbons connected conserved models that were both negatively correlated with aging in different genotypes, red ribbons connected conserved models that were both positively correlated with aging, and green ribbons connected conserved models that were oppositely correlated with aging in different genotypes. **d**, UMAP visualization of the consensus co-expression network for aging-related modules (Pearson’s correlation, *P* < 0.001 and *R*^2^ > 0.2) in muscle, neurons and hypodermis. Dots represented genes and are colored by the module they belonged to. Edges represent co-expression between genes (upper). Lower panel are heatmaps representing correlations between consensus co-expression modules and aging. Gene modules demonstrating significant correlation with aging (Pearson’s correlation, *P* < 0.001 and *R*^2^ > 0.2) in at least one genotype are shown. Bar graphs enclosed in dashed boxes represent KEGG pathways that are significantly enriched (Fisher’s exact test, Benjamini–Hochberg adjusted *P* < 0.01) for the blue module in the muscle, turquoise module in neurons, and turquoise module in the hypodermis.

Next, we focused on age-associated functional changes in different tissues. We profiled cell-type-specific Gene Ontology (GO) term enrichment changes during aging. Our analysis discovered GO terms whose enrichment levels in specific tissues showed a consistent trend of increasing or decreasing during aging (age-related GO terms) (Fig. 6a,b and Extended Data Fig. 6a,b). In neurons, all age-related GO terms undergo expression increases from the young age (day 1) to the middle age (day 6) and to the old age (day 12/14) (Fig. 6a). This includes negative regulators of axon regeneration, neuron projection regeneration and response to wounding (Fig. 6a), which may result in decreased regeneration of axon projection upon damage and neuronal dysfunction during aging. GO terms of axonal transport and cilium assembly also exhibited an age-related increase (Fig. 6a), which may be a compensatory response to the decreased axon regeneration. Interestingly, those age-associated increases did not occur in the *lipl-4 Tg* strain, except for cilium assembly (Fig. 6a). In the *daf-2(lf)* and *rsks-1(lf)* mutants, those GO terms showed a greater elevation at the middle age than in WT but did not further increase at the old age (Fig. 6a).

In contrast to neurons, the age-related GO terms exhibited a pattern of expression decrease from young to middle age and then to old age in the intestine, muscle and hypodermis (Fig. 6b and Extended Data Fig. 6a,b). The age-related GO terms specific to the intestine encompass various metabolic pathways involved in amino acid, fatty acid and carbohydrate biosynthesis and catabolism (Fig. 6b), suggesting a universal decline of metabolic processes during aging. Compared to WT, 90% of those terms show a lower degree of age-associated decrease in the three long-lived strains (Fig. 6b), suggesting improved metabolic maintenance.

Hypodermis is another key metabolic tissue in *C. elegans*, as revealed by cell-type-specific functional signatures (Fig. 2c,d). We found that, with increasing age, genes associated with the GO terms responsible for amino acid, carbohydrate derivative, glycoprotein, monocarboxylic acid and triglyceride catabolism exhibited overall decreases in expression (Extended Data Fig. 6a). This observation suggests that metabolic processes universally decline in the hypodermis with age as well. Similar age-associated decreases were observed in the *lipl-4 Tg* strain; however, many of these age-associated decreases were attenuated in the *daf-2(lf)* mutant (Extended Data Fig. 6a). In the *rsks-1(lf)* mutant, the mentioned GO terms showed expression increases at day 6 but even stronger expression decreases at day 12 (Extended Data Fig. 6a).

In the muscle, GO terms consisting of skeletal myofibril assembly, sarcomere organization, heart contraction and extracellular matrix organization showed age-associated decreases in WT (Extended Data Fig. 6b), which is consistent with muscular dysfunction and impaired physical activities in aged animals. Those decreases were suppressed in the *daf-2(lf)* mutant but not in the *lipl-4 Tg* strain or the *rsks-1(lf)* mutant (Extended Data Fig. 6b). Additionally, WT muscular cells showed age-related decreases in expression of genes responsible for the removal of superoxide radicals, which were attenuated in the *daf-2(lf), rsks-1(lf)* and *lipl-4 Tg* strains (Extended Data Fig. 6b), suggesting the antioxidant protection by the three longevity mechanisms.

Together, these findings suggest that the three longevity mechanisms alleviate age-associated functional changes in neurons and the intestine, consistent with their previously reported longevity-regulatory effects in these two tissues^5,10,12,33,34^. Furthermore, the *daf-2(lf)* mutant exerts protective effects against age-associated dysfunctions in the muscle and hypodermis. These analyses not only reveal how aging differentially impacts distinct tissues at the organism level but also uncover tissue-specific anti-aging effects conferred by different pro-longevity mechanisms.

Weighted gene co-expression network analysis (WGCNA) is another way to systemically extract tissue-specific, genotype-specific patterns during aging^35^. We first constructed co-expression networks for each tissue from different genotypes separately, to identify co-expression modules that showed significant correlation with aging, either negatively (in blue) or positively (in red), and are conserved among all genotypes (Fig. 6c and Extended Data Fig. 6c). We further linked those conserved modules between different genotypes, using a blue ribbon for negative associations with aging in both genotypes, red for both positive associations and green for the opposite (Fig. 6c and Extended Data Fig. 6c). We observed that modules negatively correlated with aging were preserved across genotypes in most tissues, whereas modules positively correlated with aging tended to be genotype specific except for neurons (Fig. 6c and Extended Data Fig. 6c). We also constructed consensus network analysis for each tissue across all genotypes, and KEGG analysis identified the enrichment of the spliceosome in several co-expression modules. However, the correlation of these modules with aging in different genotypes exhibited different trends. For example, the intestine turquoise module was negatively correlated with aging in all genotypes (Fig. 6d), whereas the muscle blue module showed a positive correlation in WT, *daf-2(lf)* and *lipl-4 Tg* worms (Extended Data Fig. 6d). For the hypodermis turquoise module, its correlation with aging was negative in WT, *daf-2(lf)* and *rsks-1(lf)* worms but positive in the *lipl-4 Tg* strain (Extended Data Fig. 6d). These results indicate that RNA processing exhibits age-related changes in a cell-type-specific and genotype-specific manner.

### Genome-wide profiling of age-related APA changes

**Extended Data Fig. 7:**
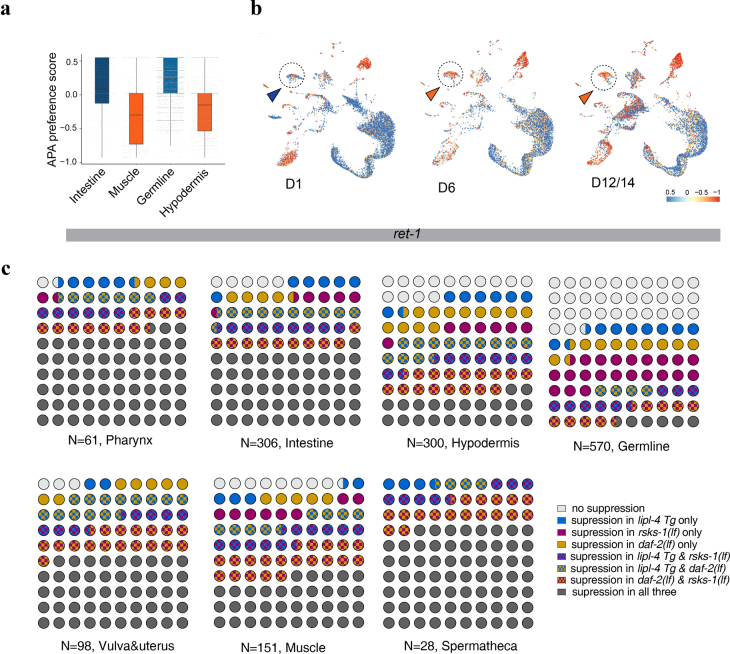
Effects of different pro-longevity strategies on age-related APA changes. **a**, The APA preference of *ret-1* across four tissues. The boxplot’s box spans the interquartile range (IQR), with the bottom and top representing the 25th and 75th percentiles, respectively, and the mean value at the middle in this case. Whiskers extend to the smallest and largest values within 1.5 times the IQR from the quartiles. **b**, UMAPs showing APA site preference of *ret-1* among all cell types at different ages. Age-related APA changes in the intestine cluster (circle) are highlighted with arrowheads (red for proximal; blue for distal). **c**, Waffle plots showing how age-related APA changes are affected by different pro-longevity mechanisms in different tissues.

To assess age-related changes in RNA processing in different tissues, we used APA as a readout. APA is a crucial RNA processing mechanism linked to alternative splicing^36,37^. For example, a previous study demonstrated that *ret-1*, encoding *C. elegans* Reticulon, exhibits two different splicing forms in muscle and intestine, and the splicing difference is reduced with aging^38^. In accordance with this previous finding, we found that *ret-1* in muscle and intestine preferentially uses proximal and distal APA sites, respectively (Extended Data Fig. 7a,b), and, as age increases, the APA preference in the intestine shifts from distal to proximal, becoming like that in the muscle (Extended Data Fig. 7a,b). Additionally, we found that, in the hypodermis and germline, *ret-1* also exhibits the opposite APA preference (Extended Data Fig. 7a,b), and, in the germline, the proportion of cells with the distal APA site is decreased with age (Extended Data Fig. 7a,b).

**Fig. 7:**
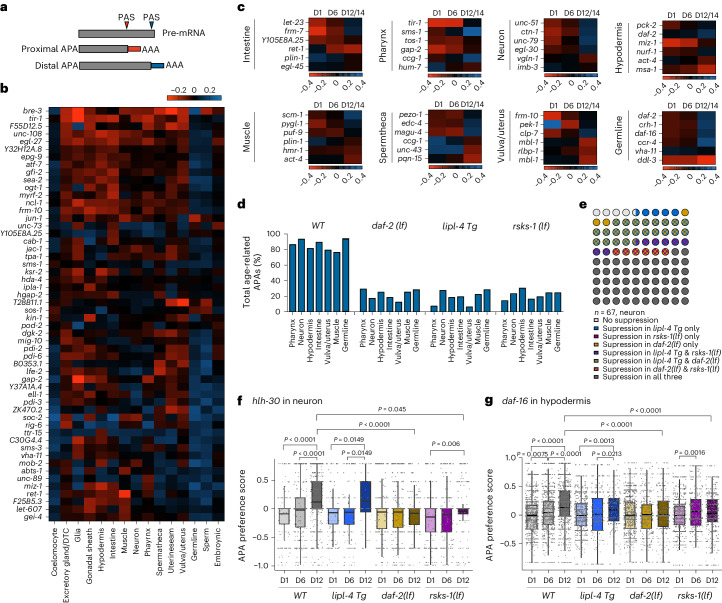
APA site usage preference across tissues and ages. **a**, Schematics of APA site preference toward the proximal or distal polyadenylation site (PAS). **b**, Heatmaps showing 55 newly identified genes with tissue-specific preference for APA sites. **c**, For each tissue, six examples of genes exhibiting age-related changes in APA site usage are shown. **d**, Percentage of genes showing an age-related shift toward the distal APA usage is decreased in different long-lived strains. **e**, Waffle plots showing how age-related APA changes in neurons are differently affected in the three long-lived strains. Total gene number = 67. **f**, APA site preference shifts of *hlh-30* in neurons from day 1 to day 12 are suppressed in the *rsks-1(lf)* and *daf-2(lf)* but not the *lipl-4 Tg* long-lived strains. ****P < 0.0001, ***P < 0.001 and NS *P* > 0.05 by two-sided Wilcoxon rank-sum test with Dunn–Sidak correction. **g**, APA site preference shifts of *daf-16* in the hypodermis from day 1 to day 12 are completely suppressed in the *daf-2(lf)* mutant and partially in the *rsks-1(lf)* mutant. NS *P* > 0.05; *P* value by two-sided Wilcoxon rank-sum test with Dunn–Sidak correction. For **f** and **g**, the box plot’s box spans the IQR, with the bottom and top representing the 25th and 75th percentiles, respectively, and the mean value at the middle in this case. Whiskers extend to the smallest and largest values within 1.5 times the IQR from the quartiles.

We then applied the polyApipe tool to systemically profile APA types, proximal versus distal adenylation site in pre-mRNAs (Fig. 7a). This analysis identified 851 candidate genes that showed a tissue-specific APA preference (Supplementary Table 3), and 55 genes with expression in over 20% of cells were highlighted (Fig. 7b). We further searched for genes displaying age-related APA changes and identified hundreds of candidates for each tissue (Fig. 7c), with six examples shown for each tissue (Supplementary Table 3). Interestingly, among these candidates that exhibited age-related APA changes in different tissues, over 70% of them increased the utilization of the distal APA sites from day 1 to day 12/14 (Fig. 7d). It is known that APA preference is regulated by changes in cell proliferation and differentiation, and increased distal usage is often associated with cell differentiation^37^. In adult *C. elegans*, all somatic cell types are post-mitotic. Thus, although the distal APA increase in germ cells might be associated with a decreased capacity for cell proliferation, the alterations in somatic tissues are unlikely to be related to changes in cell proliferation or differentiation. Instead, they suggest that APA can be globally regulated by aging.

### Unveiling tissue-specific regulation of APA by pro-longevity strategies

Next, we investigated whether and how pro-longevity mechanisms affect age-related APA changes. We found that the age-related increase in the utilization of the distal APA sites was overall suppressed in the long-lived strains (Fig. 7d). Furthermore, most age-related APA changes were suppressed by at least one pro-longevity mechanism, with suppression rates over 85% in somatic tissues and 68% in the germline (Fig. 7e, Extended Data Fig. 7c and Supplementary Table 3). For each specific gene, the suppression pattern exhibited differences in the distinct long-lived strains. For example, *hlh-30* encodes the TFEB TF, which is required for the lifespan extension in the *daf-2(lf)* and *rsks-1(lf)* mutants^39^. We found that the distal APA usage of *hlh-30* is increased during aging in neurons of WT and the *lipl-4 Tg* worms (Fig. 7f). This age-related increase did not occur in the *daf-2(lf)* mutant (Fig. 7f) and was partially suppressed in the *rsks-1(lf)* mutant (Fig. 7f). In another example, *daf-16* encodes the FOXO TF required for the longevity effect caused by the reduction in the IIS and mTOR signaling^9,32,40^. We found that *daf-16* exhibits an age-related increase in the distal APA usage in hypodermal cells of WT worms, which was completely absent in the *daf-2(lf)* mutant (Fig. 7g). This increase on day 12 was suppressed in the *rsks-1(lf)* mutant but not in the *lipl-4 Tg* strain (Fig. 7g). The distal APA preference is associated with longer 3′ untranslated region (UTR), which is known to decrease mRNA stability and translational efficiency^41,42^. Thus, suppressing the increased distal APA usage in the long-lived strains may help maintain the protein output of HLH-30 and DAF-16 during aging, contributing to the longevity-promoting effect.

## Discussion

Our studies present not only adult aging cell atlases of WT *C. elegans* but also a single-cell transcriptomic database for three longevity-promoting strains. Together with aging atlases established in other species, including human, mouse and fruit fly^25,43–48^, crucial insights are provided into aging-related molecular changes at a single-cell level, greatly enhancing understanding of the cellular aging process in the context of whole organisms. Interestingly, in addition to well-known cell clusters in adult *C. elegans*, we observed a cell cluster that increases in number with age in all genotypes, marked by the expression of stress response genes. We named this group age-related stressed cells (ARSC), which bear certain resemblance to stressed neurons described in the previous study^17^. Further research is required to determine whether these cells result from procedural damage during sample preparation or reflect profound transcriptomic dysregulation during aging.

This dataset offers an opportunity to comprehensively investigate the somatic and reproductive aging process. Machine-learning-based tissue-specific transcriptomic aging clocks pave the way for assessing the anti-aging effect of different pro-longevity interventions in different tissues and gaining molecular insights into these tissue-specific effects. Among the tissues analyzed, neurons stand out as particularly significant. They not only exhibit a heightened sensitivity to age-related alterations but also benefit substantially from different pro-longevity interventions. This finding is consistent with previous works from *C. elegans* to mammals, which demonstrate the key role of neurons in regulating aging and longevity^49^. This study built the first germline-specific trajectory map in *C. elegans*. Our findings reveal that the numbers of germline nuclei in the transition zone and meiosis region decrease with increasing age, which was not reported before. Additionally, in line with previous findings^50^, we observed a decrease in the numbers of GSCs and mitotic cells during aging; however, this decrease did not reach statistical significance. It is interesting to note that transcriptomic aging in the germline continues to progress even after reproductive cessation, which reveals age-related decreases in translation, organelle biogenesis and protein quality control along different germ cell stages.

APA plays a crucial role in the control of mRNA metabolism, gene regulation and protein diversification^51^. Our study provides, to our knowledge, the first systematic profiling of APA changes at the whole transcriptome level. Interestingly, APA events exhibit tissue-specific distribution, undergo significant changes during aging and can be differentially regulated by different pro-longevity mechanisms. We discovered that, during aging, all cell types shift their APA preference toward the distal site, and this shifted preference is suppressed in the long-lived strains. Previous studies revealed that the usage of the distal APA site is inversely correlated with the level of core polyadenylation factors^37^. Additionally, the increased usage of distal APA sites may lead to longer 3′ UTRs, which are associated with mRNA instability^41,42^. Based on our findings, we speculate that, with aging, the level of core polyadenylation factors may decrease and the length of 3′ UTRs may increase, potentially resulting in declines in translational efficiency. Thus, suppressing the distal APA usage in the long-lived strains may help improve protein outputs, contributing to their longevity effects.

## Methods

### *C. elegans*, bacteria strains and maintenance

The following strains were used in this study: N2, CB1370 *daf-2(e1370)*, RB1206 *rsks-1(ok1255)* and MCW14 *raxIs3 [ges-1p::lipl-4::SL2GFP]*. CB1370 and MCW14 were outcrossed to N2 for eight times. RB1206 was outcrossed to N2 for six times. The strains N2, CB1370 and RB1206 were obtained from the Caenorhabditis Genetics Center (CGC). MCW14 was generated in our laboratory. All strains were incubated at 20 °C for both maintenance and experiments. *Escherichia coli* OP50 and HT115 were obtained from the CGC. *E. coli* BW25113, Δlon is from the *E. coli* Keio collection, courtesy of Christophe Herman (Baylor College of Medicine).

### Worm preparation

Worms were synchronized using a bleach-based egg isolation method and subsequently starved in M9 buffer at the L1 stage for 24 h. Worms of the desired genotype were then seeded to NGM plates pre-seeded with *E. coli* OP50. Approximately 3,000–5,000 *C. elegans* were washed off the plates at day 1, day 6, day 12 or day 14. To keep all the cell types, including the germline, we used worms that were actively reproducing. For the maintenance of aged worms, we transferred the worms every day by washing them off the plate with M9 buffer and filtering with 40-μm strainers (pluriStrainer, SKU 43-10040-40 and SKU 43-50040-51) to isolate the adult worms while discarding the larvae and eggs in the flow through M9 buffer.

### Nuclei isolation

Worms were washed three times with PBS and collected in a 1.5-ml tube. We then added 100 μl of homogenization buffer^23^ and ground the worms with a pestle motor for 30 s on ice. To minimize nuclei adhesion on the surface, all the pestles, tubes and filters were pre-coated with a homogenization buffer or 1× PBS. Then, 900 μl of homogenization buffer was added to wash the pestle, and the total 1 ml of homogenized sample was transferred into a 1-ml Dounce tissue grinder (Wheaton, 357538). The grinder was autoclaved overnight in a 220 °C oven to deactivate ribonuclease. After placing the grinder on ice, 20 strokes were applied using a loose pestle, followed by another 20 strokes using a tight pestle, while avoiding generating foams. The 1,000-μl samples were filtered through a cell strainer (35 μm) and then through a Flowmi cell strainer (Bel-Art, H13680-0040) into a new 1.5-ml tube. The tubes were centrifuged for 10 min at 1,000*g* at 4 °C, and the supernatant was removed while not disturbing the pellet, which was hardly visible. The pellet was resuspended with 500 μl of 1× PBS with 0.5% BSA and RNAase inhibitor and filtered with a Flowmi cell strainer (Bel-Art, H13680-0040) again into a 5-ml flow cytometer tube. A total of 20 μl was transferred into another flow cytometer tube and diluted with 180 μl of 1× PBS with 0.5% BSA and RNAase inhibitor as an unstained control. Unstained control is mandatory for each sorting experiment to guarantee correct nuclei gating even when you perform the sorting experiment routinely. Hoechst (Invitrogen, 33342) was used to stain the nuclei in a 1:1,000 working concentration. The staining is fast as the nuclei are fluorescent within minutes, and there is no need to wash out the dye. We coated the 1.5-ml sorting collection tube with 1× PBS with 0.5% BSA and RNAase inhibitor, and approximately 300,000 nuclei were sorted with Hoechst 33342 positive gating, which indicates DNA content, and forward scatter area (FSC-A) gating, representing the particle size above threshold. The intestinal nuclei, which are polypoid (32 N), usually form an obvious cluster separating from the other 2 N somatic nuclei, and these were also included in our collection. The collection tube was centrifuged at 800*g* for 8 min at 4 °C, and the sheath buffer supernatant was carefully removed before resuspending the sorted nuclei with 40–50 μl of 1× PBS with 0.5% BSA and RNAase inhibitor. The concentration and morphology of the nuclei were checked under a microscope to ensure high-quality nuclei isolation. If the results were desirable, we proceeded to generate gel emulsion with a 10x Chromium Controller.

For sorting the nuclei, we used either a BD LSR II or a Sony MA800 sorter. To obtain the best nuclei morphology and the least RNA degradation, it is recommended to minimize the sorting time.

### snRNA-seq

The nuclei suspensions were loaded onto the 10x Chromium Controller, and library preparation was carried out using the published 10x Chromium Single Cell 3′ v2/v3 Solution protocol (single/dual index). The resulting libraries were sequenced on HiSeq 4000 or NovaSeq 6000 platforms, with a depth ranging from 6,306 to 29,862 reads per cell, with the recommended cycle numbers: 26 cycles for read 1, eight cycles for i7 index and 98 cycles for read 2.

### snRNA-seq data pre-processing

We used Cell Ranger (6.0, 10x Genomics) to align raw base call (BCL) sequence files or FASTQ files to the *C. elegans* genome (WS282, WormBase) and generated feature-barcode matrices. Doublets were removed based on the recommended ratio provided by the 10x Genomics Chromium Next GEM Single Cell 3′ Reagent Kits v3.1 user guide (CG000204 Rev D), with a score calculated by DoubletFinder (https://github.com/chris-mcginnis-ucsf). The feature-barcode matrices for each sample were constructed into a Seurat (R package, 4.0.5) object for downstream analysis. Cells were filtered by requiring a minimum of 100 genes to be expressed, and genes were filtered by being expressed in at least three cells. Integration of samples was performed with the Seurat canonical correlation analysis (CCA) method to remove the batch effects. We tested CCA, reciprocal principal component analysis and Harmony (0.1) for integration and chose the method with the best performance. The top 2,000 variable features were used for integration anchor identification. Uniform manifold approximation and projection (UMAP) and t-distributed stochastic neighbor embedding dimension reduction were performed with the first 50 dimensions from principal component analysis. Clustering was performed at multiple Leiden resolutions.

### Cell type annotation, marker identification and subclustering

We used two approaches to assign cell types to clusters and subclusters. The first approach involved identifying a set of marker genes specific to each cluster using the FindClusters function in Seurat. Then, we compared these marker genes of each cluster to previously reported microscopy-based expression profiles in the literature to confirm the cell type assignment and also used the enrichment analysis tool in WormBase^52^. The second approach used the SingleR^53^ package to perform automatic annotation by comparing the expression profiles of the cells to those of the reference datasets, enabling accurate annotation of cell types. The reference datasets used in this study include refs. ^17,18^. The marker gene of each tissue was identified by an accurate and fast cosine similarity-based method, COSG^54^, with default settings.

To further dissect the heterogeneity within tissues, we subsetted the Seurat object to include only cells of interest and identified highly variable genes. The data were then subjected to CCA integration and dimensionality reduction followed by clustering to obtain subclusters. The subclusters were then annotated using a similar approach to the main clusters. The known marker gene for reference is summarized in Supplementary Table 1. For the neuron subclustering, giving the substantial number of subclusters and a good correlation of our neuron clusters with previous dataset^17^, we rely mostly on the reference-based mapping method for annotating the neuron types.

### Cell-type-specific functional signature analysis

We employed the R package AUcell (version 1.20.1) to calculate the enrichment score of a set of genes in individual single cells for cell-type-specific functional features analysis. AUCell outputs an enrichment score by calculating the area under the curve to determine whether the predefined gene set is significantly enriched among all the genes expressed in each cell. To quantify the enrichment of functional signatures, we computed the median AUCell scores for these gene sets across all single cells from a specific tissue in day 1 WT worms. For each tissue, we selected the features with the highest AUCell scores within that tissue. These features were then included as representative elements in a heatmap. In the heatmap, each feature is represented with z-scores to ensure accurate and standardized comparisons.

Gene set annotations were extracted from wormEnrichr^55,56^, including InterPro classification of protein families (176 gene sets), KEGG pathway networks (111 gene sets), regulatory interactions that connect TFs and the genes that these factors putatively regulate based on DNA binding site motifs (59 gene sets) and GO libraries for biological process (1,711 gene sets). The same analyses were conducted using published data with matching tissue annotations, allowing for a direct comparison of cell-type-specific functional signatures with our dataset.

### Cell-type-specific differential expression analysis

We used the Seurat function FindMarkers to perform differential expression analysis between groups of cells. This function employed a non-parametric Wilcoxon rank-sum test to identify genes that exhibited differential expression between the groups. Genes with an adjusted *P* value less than 0.05 were considered differentially expressed.

### Germline trajectory

To investigate the germline trajectory in our scRNA-seq data, we performed a series of analyses using various tools. First, germline cells were re-integrated and clustered at a Leiden resolution of 0.5 using Seurat. Two clusters with a sparse UMAP distribution were identified as intra-tissue doublets and removed. Next, we used Slingshot with default settings, using the cluster as label inputs and UMAP embeddings as reduced dimension input, to construct cell lineages and recover pseudotime. As a result, we identified two major trajectories and focused our analysis on the trajectory that ended at oocytes.

For the analysis of gene expression patterns along trajectories and for comparing them across genotypes, we applied the figGAM function from the R package TradeSeq. This package uses a generalized additive model to fit the expression levels of each gene in each cell and further estimate the nonlinear relationship between gene expression and cellular trajectory.

The conditionTest function evaluates differential expression patterns between conditions by calculating the Wald distance for gene changes in pairwise comparisons.

### Velocity/PAGA

To investigate the developmental trajectory of germline cells, we performed a range of analyses using scVelo (0.2.4) and CellRank (1.5.1). The processed germline Seurat object was converted to an AnnData object, and the top 5,000 highly variable genes were selected for velocity calculation. We used the first 30 principal components and set the neighbor number to 50 to perform the velocity analysis. This approach allowed us to gain insights into the changes in gene expression over time and identify the major differentiation pathways and cellular lineages.

In addition to the velocity analysis, we used CellRank to perform PAGA analysis with weight of connectivities set to 0.3. This method summarizes the connectivity between the clusters of cells and identifies the major cellular lineages and terminal states.

### Aging clocks

Aging clocks are machine learning models designed to predict the age of input cells. We trained these models with log-normalized expression data from BootstrapCells^57^ and the true chronological age of WT worms at different timepoints. The R package glmnet (version 4.1-3) was employed to fit the LASSO regression models via penalized maximum likelihood. Parameters were optimized and BootstrapCells were generated following the methods described by Buckley et al.^57^. Specifically, the transcriptomes of 15 single cells were randomly sampled without replacement from the pool of cells of a given tissue, and gene counts were then summed. This bootstrapping process was repeated 100 times for each tissue. The resulting expression profiles of the BootstrapCells were log normalized as ln((gene transcripts / cell transcripts) × 10,000) and served as input for training the tissue-specific aging clocks. Aging clocks are trained on tissues with more than 50 cells. In our trained aging clocks, genes with non-zero coefficients are designated as aging clock genes. These coefficients represent their relative contributions in predicting age within each specific tissue. Genes assigned positive coefficients represent their higher expression levels and are correlated with an older age in the worms. Conversely, genes with negative coefficients suggest an inverse relationship, where lower expression levels are associated with younger ages.

For the validation of the aging clocks, we performed leave-one-batch-out cross-validations. This involved excluding one biological replicate during the training phase and reserving it as a test dataset. This approach was to ensure that no cells from the same worm were present in both the training data and the test dataset, thereby eliminating the risk of performance inflation due to data overlap. After training the models, we applied the models to predict the ages of the withheld test samples to evaluate the performance of the trained models. We repeated this for all four timepoints.

We quantified the performance of the models in a similar way as described in Buckley et al.^57^. Specifically, the data were presented as a correlation between the actual chronological age of the worm from which the cell originated and the predicted chronological age (median as red dot) for the reserved test dataset. We fitted a linear model (blue line) through the points as well as the 95% confidence interval (light gray) using geom_smooth (ggplot2). The square of Pearson’s correlation (*R*^2^) is indicated on the graph.

We used a published *C. elegans* scRNA-seq dataset^21^ for external validation of our tissue-specific aging clocks. The annotations of four somatic tissues, including neurons, hypodermis, intestine and muscle, were matched between the two studies, and we predicted the chronological age with trained aging clocks based on all WT worms in our datasets. The evaluation data were presented as a correlation between the actual chronological age of the worm from which the cell originated based on published data and the predicted chronological age based on our aging clocks.

### scMMD

scMMD is a computational method used to analyze the differences between cell populations in single-cell data. It uses the MMD metric to calculate the extent of divergence between two cell populations and a *P* value. scMMD is an open-source Python package available on GitHub (https://github.com/calico/scmmd).

To perform the analysis, scMMD applies a bootstrap resampling approach with a sample size of 15, running 300 iterations to ensure the robustness and reliability of the results.

### Co-expression network analysis

We performed co-expression network analysis with WGCNA. The R package hdWGCNA (version 0.2.03) was used, which is specifically designed to perform co-expression network analysis on single-cell data. We first constructed metacells and selected soft power thresholds based on the manual guidance. We performed consensus network analysis for each tissue across all the strains. A consensus network can be used to construct a unified network for all the strains and identify networks that are conserved across different genotypes. We subsequently assigned genes to modules based on constructed co-expression networks and set chronological age as a trait to calculate module–trait correlation and identify aging-related modules in different strains. Modules with *P* < 0.001 and *R*^2^ > 0.2 were defined as positively correlated with aging, whereas modules with *P* < 0.001 and *R*^2^ < −0.2 were defined as negatively correlated with aging. Genes in consensus modules that were correlated with aging in at least one strain were extracted. We also separately constructed tissue-specific co-expression networks for all the strains to identify genotype-specific aging-related modules. We tested the conservation between all the modules from different genotypes with Fisherʼs exact test. Modules with *P* values less than 0.01 were considered conserved modules between genotypes and are linked in the circos plot.

### APA analysis

To analyze APA in our 10x Genomics scRNA-seq dataset, we used the R package polyApipe with default settings. We used the *C. elegans* genome (WS282) to generate APA site references for the analysis.

To investigate age-related APA site preference changes in WT worms, we combined the day 12 and day 14 samples owing to cell number limitations. We then performed a comparison among day 1, day 6 and day 12/14, selecting genes that were expressed in at least 10% of the cells in all ages. This analysis allowed us to identify genes with age-related changes in APA site preference, providing insights into the molecular mechanisms underlying aging in *C. elegans*.

### Age-related and tissue-specific changes in GO terms enrichment

We identified tissue-specific GO terms in WT day 1 worms. We first calculated AUCell scores for each GO term across all single cells from day 1 WT worms. Subsequently, these GO terms were assigned as tissue specific based on the tissue exhibiting the highest median AUCell score. We then calculated the AUcell scores for cells from the corresponding tissue in WT worms at different ages: day 1 (young age), day 6 (middle age) and days 12 and 14 (old age). For each age group, we determined the median AUCell scores for these tissue-specific GO terms. Tissue-specific GO terms that exhibited a consistent trend of at least a 20% change, either increasing or decreasing, during the aging process were classified as age related. The significance level for comparing AUCell scores between each age group was set at 0.01.

### Tissue-specific age prediction for long-lived strains

We employed tissue-specific aging clocks, which were trained on the expression data of corresponding tissues from WT, to predict the biological age of tissues in long-lived strains. To generate the input for the aging clocks, we generated BootstrapCells for each tissue in the long-lived strains. Then, log-normalized expression data from BootstrapCells for each tissue were used as input of the trained tissue-specific aging clocks to predict the biological age of tissues for long-lived strains. One-sample *t*-test was performed to assess the significance level of the differences between the predicted biological age and the true chronological age of the tissues in long-lived strains.

### Lifespan and survival rate measurements

To synchronize the age of worms, a bleach-based egg isolation method was used, followed by at least 24 h of starvation in M9 buffer at the L1 developmental stage. To ensure experimental rigor, all genotypes and conditions were tested in parallel. The synchronized L1 worms were then allowed to grow until they reached the first day of adulthood, at which point they were transferred to new plates every 2 d. In total, approximately 100 animals were analyzed for each condition and genotype, with 30–50 animals per 6-cm plate. Death was determined by the complete cessation of movement in response to gentle mechanical stimulation.

### Statistics and reproducibility

No statistical methods were used to pre-determine sample sizes, but our sample sizes are similar to those reported in previous publications^21,23,25^. No randomization method was used in any of the experiments. Data collection and analysis were not performed blinded to the conditions of the experiments. Statistical analyses for the lifespan and individual genes’ APA usage in longevity models were conducted using SPSS23 (IBM), and the lifespan curve was generated using GraphPad Prism 9. The Mann–Whitney–Wilcoxon test was employed to calculate the differences in APA site preference between cell populations, allowing for the identification of statistically significant differences. To identify tissue-specific APA site preference, we used a criterion of false discovery rate-adjusted *P* < 0.05 in any inter-tissue comparison (for example, neuron–muscle) and a gene expression level of at least 20% in the tissue cells. This approach allowed us to identify genes with differential APA site preferences between different tissues at a substantial expression level. Statistical analyses for the other figures were conducted in Python or R with the corresponding statistical test.

### Software used for data processing and analysis

Software used for data processing and analysis was as follows: Cell Ranger (https://support.10xgenomics.com/single-cell-gene-expression/software), Seurat (4.0.5)^58^, BD FACSDiva (9.0.1), Sony Cell Sorter (2.1.6), SingleR (1.8.1)^53^, AUcell (1.20.1)^59^, scMMD (1.0)^60^, Slingshot (1.8.0)^27^, Tradeseq (1.12.0)^61^, scVelo (0.2.4)^28^, CellRank (1.5.1)^62^, circlize (0.4.15)^63^, DoubletFinder (2.0)^64^, glmnet (4.1-3)^65^, hdWGCNA (0.2.03)^66^, ggplot2 (3.3.5)^67^, COSG (0.9.0)^54^, polyApipe (1.0) (https://github.com/MonashBioinformaticsPlatform/polyApipe), GraphPad Prism 9 (https://www.graphpad.com/scientific-software/prism/) and SPSS23.

### Reporting summary

Further information on research design is available in the Nature Portfolio Reporting Summary linked to this article.

### Supplementary information

Reporting Summary

Supplementary Code 1 Some of the codes used in the analysis.

Supplementary Table 1 Reference marker genes in tissue subclustering and sample information.

Supplementary Table 2 Aging clock genes of different major tissues and top 621 genes showing age-related changes in germline temporal patterns.

Supplementary Table 3 IIntegrative analysis of genes with tissue-specific and age-related APA variations and age-related APA changes suppressed by longevity mechanisms.

## Data Availability

We provide an interactive website (http://mengwanglab.org/atlas) for exploring the code and our data, including all cells, gene expression, subclusters and APA site preferences. Raw FASTQ sequencing files, expression matrix, Seurat objects and other analysis outputs can be downloaded from the Gene Expression Omnibus under accession number GSE229022.

## Code availability

The code used for some of the analyses is included in the Supplementary Information and is deposited on GitHub at https://github.com/fulabgenomics/CAWA to share with the community.
