# Supplementary material for: Aging atlas reveals cell-type-specific effects of pro-longevity strategies
Source: Nat Aging. 2024 May 30;4(7):998–1013. doi: 10.1038/s43587-024-00631-1 (PMC11257944; doi:10.1038/s43587-024-00631-1)
Supplement: Supplementary file 1 — Reporting Summary [file 43587_2024_631_MOESM1_ESM.pdf]

nature portfolio

Corresponding author(s): Meng Wang; Yusi Fu; Hongjie Li

Last updated by author(s): Mar 21, 2024

Reporting Summary

Nature Portfolio wishes to improve the reproducibility of the work that we publish. This form provides structure for consistency and transparency in reporting. For further information on Nature Portfolio policies, see our Editorial Policies and the Editorial Policy Checklist.

Statistics

For all statistical analyses, confirm that the following items are present in the figure legend, table legend, main text, or Methods section.

n/a	Confirmed	
☐	☒	The exact sample size (n) for each experimental group/condition, given as a discrete number and unit of measurement
☐	☒	A statement on whether measurements were taken from distinct samples or whether the same sample was measured repeatedly
☐	☒	The statistical test(s) used AND whether they are one- or two-sided<br>*Only common tests should be described solely by name; describe more complex techniques in the Methods section.*
☒	☐	A description of all covariates tested
☐	☒	A description of any assumptions or corrections, such as tests of normality and adjustment for multiple comparisons
☐	☒	A full description of the statistical parameters including central tendency (e.g. means) or other basic estimates (e.g. regression coefficient) AND variation (e.g. standard deviation) or associated estimates of uncertainty (e.g. confidence intervals)
☐	☒	For null hypothesis testing, the test statistic (e.g. F, t, r) with confidence intervals, effect sizes, degrees of freedom and P value noted<br>*Give P values as exact values whenever suitable.*
☒	☐	For Bayesian analysis, information on the choice of priors and Markov chain Monte Carlo settings
☒	☐	For hierarchical and complex designs, identification of the appropriate level for tests and full reporting of outcomes
☒	☐	Estimates of effect sizes (e.g. Cohen's d, Pearson's r), indicating how they were calculated

Our web collection on statistics for biologists contains articles on many of the points above.

Software and code

Policy information about availability of computer code

Data collection	BD FACSDiva Software Diva Version 9.0.1 and Sony Cell Sorter Software Version 2.2.6
Data analysis	Cell Ranger(6.0.1)<br>Seurat (4.0.5)<br>SingleR (1.8.1)<br>AUcell (1.20.1)<br>scMMD (1.0)<br>Slingshot (1.8.0)<br>Tradeseq (1.12.0)<br>scVelo (0.2.4)<br>Cellrank (1.5.1)<br>circlize (0.4.15)<br>Doubletfinder (2.0)<br>glmnet (4.1-3)<br>hdWGCNA (0.2.03)<br>ggplot2 (3.3.5)<br>polyApipe (1.0)<br>Prism 9<br>SPSS23

For manuscripts utilizing custom algorithms or software that are central to the research but not yet described in published literature, software must be made available to editors and reviewers. We strongly encourage code deposition in a community repository (e.g. GitHub). See the Nature Portfolio guidelines for submitting code & software for further information.

Data

Policy information about availability of data

All manuscripts must include a data availability statement. This statement should provide the following information, where applicable:

- Accession codes, unique identifiers, or web links for publicly available datasets
- A description of any restrictions on data availability
- For clinical datasets or third party data, please ensure that the statement adheres to our policy

We make our dataset,both raw and processed, available to the public through GEO database (GSE229022) and confirmed that it was made public.The code used for some of analyses is included on our website to share with the community.

Research involving human participants, their data, or biological material

Policy information about studies with human participants or human data. See also policy information about sex, gender (identity/presentation), and sexual orientation and race, ethnicity and racism.

Reporting on sex and gender	N.A.
Reporting on race, ethnicity, or other socially relevant groupings	N.A.
Population characteristics	N.A.
Recruitment	N.A.
Ethics oversight	N.A.

Note that full information on the approval of the study protocol must also be provided in the manuscript.

Field-specific reporting

Please select the one below that is the best fit for your research. If you are not sure, read the appropriate sections before making your selection.

☒ Life sciences ☐ Behavioural & social sciences ☐ Ecological, evolutionary & environmental sciences

For a reference copy of the document with all sections, see nature.com/documents/nr-reporting-summary-flat.pdf

Life sciences study design

All studies must disclose on these points even when the disclosure is negative.

Sample size	We did not perform a sample size calculation. Instead, we performed the 10x sequencing aiming at each sample for 10,000 cells. There are some variation of cell number in each sample. The sample size is limited by the current methodology of 10x Chromium sequencing.No statistical methods were used to pre-determine sample sizes but our sample sizes are similar to those reported in previous publications
Data exclusions	Cells are filtered through DoubletFinder score for doublet removal, and cells with low UMI are removed from analysis.
Replication	Wild-type D1, D6, D12, D14 worms have 3 replicates of snRNAseq. Other samples don't have replicates due to limitation of budget and manpower.
Randomization	Given each single nuclei sequencing run was from nuclei of thousands of worms, randomization of animal is not needed.
Blinding	Data collection and analysis were not performed blind to the conditions of the experiments.

Reporting for specific materials, systems and methods

We require information from authors about some types of materials, experimental systems and methods used in many studies. Here, indicate whether each material, system or method listed is relevant to your study. If you are not sure if a list item applies to your research, read the appropriate section before selecting a response.

Materials & experimental systems

n/a	Involved in the study
☒	☐ Antibodies
☒	☐ Eukaryotic cell lines
☒	☐ Palaeontology and archaeology
☐	☒ Animals and other organisms
☒	☐ Clinical data
☒	☐ Dual use research of concern
☒	☐ Plants

Methods

n/a	Involved in the study
☒	☐ ChIP-seq
☐	☒ Flow cytometry
☒	☐ MRI-based neuroimaging

Animals and other research organisms

Policy information about studies involving animals; ARRIVE guidelines recommended for reporting animal research, and Sex and Gender in Research

Laboratory animals	C elegans strains: N2, CB1370 daf-2(e1370), RB1206 rsks-1(ok1255), and MCW14 raxIs3 [ges-1p::lipl-4::SL2GFP]. CB1370 and MCW14 has been outcrossed to N2 for 8 times. RB1206 outcrossed to N2 for 6 times. The strains N2, CB1370, and RB1206 were obtained from the Caenorhabditis Genetics Center (CGC). MCW14 was generated in our lab
Wild animals	N.A.
Reporting on sex	C. elegans is naturally hermaphrodite. We use hermaphrodite worms for our experiments.
Field-collected samples	N.A.
Ethics oversight	No ethics oversight needed for worms. (Johnson TE (2003) Advantages and disadvantages of Caenorhabditis elegans for aging research. Exp Gerontol 38:1329–1332)

Note that full information on the approval of the study protocol must also be provided in the manuscript.

Flow Cytometry

Plots

Confirm that:

☒ The axis labels state the marker and fluorochrome used (e.g. CD4-FITC).

☒ The axis scales are clearly visible. Include numbers along axes only for bottom left plot of group (a 'group' is an analysis of identical markers).

☒ All plots are contour plots with outliers or pseudocolor plots.

☒ A numerical value for number of cells or percentage (with statistics) is provided.

Methodology

Sample preparation	To prepare the worms for analysis, they were washed three times with PBS and collected in a 1.5 mL tube. We added 100 µl of homogenization buffer and ground the worms with a pestle motor for 30 seconds on ice. To prevent nuclei adhesion on the surface, all pestles, tubes, and filters were pre-coated with a homogenization buffer or 1x PBS. We added 900 µl of homogenization buffer to wash the pestle, and the total 1 mL homogenized sample was transferred into a 1 mL Dounce tissue grinder (Wheaton 357538) that had been sterilized overnight at 220 degree to deactivate ribonuclease. After placing the grinder on ice, we applied 20 strokes using a loose pestle, followed by another 20 strokes using a tight pestle, while avoiding generating foams. The 1000 µl samples were filtered through a cell strainer (35 µm) and then through a Flowmi cell strainer (BelArt, H13680-0040) into a new 1.5 mL tube. The tubes were centrifuged for 10 minutes at 1000 g at 4 degree, and the supernatant was removed without disturbing the pellet, which was hardly visible. The pellet was resuspended with 500 µl 1x PBS with 0.5% BSA and RNAase inhibitor and filtered with a Flowmi cell strainer (BelArt, H13680-0040) again into a 5 mL flow cytometer tube. We transferred 20 µl of the sample into another flow cytometer tube and diluted it with 180 µl 1x PBS with 0.5% BSA and RNAase inhibitor as an unstained control. Hoechst (Invitrogen 33342) was used to stain the nuclei in a 1:1000 working concentration. We coated the 1.5 mL sorting collection tube with 1x PBS with 0.5% BSA and RNAase inhibitor and sorted ~300,000 nuclei with Hoechst 33342 positive gating, which indicates DNA content, and forward scatter area (FSC-A) gating, representing the particle size above threshold. The intestinal nuclei, which are polypoid (32N) and usually form an obvious cluster separating from the other 2N somatic nuclei, were also included in our collection. The collection tube was centrifuged at 800 g for 8 minutes at 4 degree and the sheath buffer supernatant was carefully removed before resuspending the sorted nuclei with 40-50 µl 1x PBS with 0.5% BSA and RNAase inhibitor. We checked the concentration and morphology of the nuclei under a microscope to ensure high-quality nuclei isolation. If the results were desirable, we proceeded to generate a gel emulsion with 10X Chromium Controller.
Instrument	DB LSR II or Sony MA800 sorter

Software	BD FACSDiva Software Diva Version 9.0.1 and Sony Cell Sorter Software Version 2.2.6
Cell population abundance	The purity of the samples are >90%. Assessed by re-sorting.
Gating strategy	Gated by fluorescence of Hoechst dye

☒ Tick this box to confirm that a figure exemplifying the gating strategy is provided in the Supplementary Information.
